# Establishment and application of nano-liposomes system for targeted therapy of methicillin-resistant *Staphylococcus aureus* enteritis

**DOI:** 10.1016/j.mtbio.2025.102380

**Published:** 2025-10-07

**Authors:** Qingli Yang, Jindi Wang, Pengdong Sun, Fangyuan Zhao, Jian Ju

**Affiliations:** a Special Food Research Institute, Qingdao Agricultural University, Qingdao, 266109, People's Republic of China; b Qingdao Special Food Research Institute, Qingdao, 266109, People's Republic of China; c Key Laboratory of Special Food Processing (Co-construction by Ministry and Province), Ministry of Agriculture Rural Affairs, People's Republic of China; d Shandong Technology Innovation Center of Special Food, Qingdao, 266109, People's Republic of China

**Keywords:** Resveratrol, Nisin, Nanoliposomes, MRSA enteritis, Targeted therapy

## Abstract

Inspired by the damage caused by bacterial pore-forming toxins to cell membranes, this study utilized the film-ultrasound dispersion method and polyethylene glycol (PEG) modification to prepare resveratrol and nisin-loaded nanoliposomes (RN-NPs) using soy lecithin as the main encapsulating material. These RN-NPs were designed for targeted therapy against Methicillin-resistant *Staphylococcus aureus* (MRSA) enteritis. The synergistic inhibitory effects of resveratrol and nisin (RN) on MRSA were confirmed through SEM, FCM, and gel electrophoresis. In addition to being highly stable and resistant to erosion by stomach acid, RN-NPs can also be specifically released to treat MRSA enteritis. In animal experiments, RN-NPs exhibited superior therapeutic effects against MRSA enteritis compared to free RN and positive drugs. They showed a considerable improvement in pharmacodynamic indicators such as intestinal cytokine expression, MPO activity, and histological evaluation scores. Consequently, RN-NPs have been proven to be a very promising targeted drug delivery system for the management of *MRSA* enteritis. This study has important guiding significance for the future development of targeted drug delivery systems for enteritis.

## Highlights

• Inspired by pore-forming toxins, a strategy for passive targeted treatment of methicillin-resistant *S. aureus* (MRSA) enteritis was proposed, which provided a potential clinical application scheme for targeted treatment of methicillin-resistant *S. aureus* enteritis.
• A nanoliposome containing Nisin and resveratrol (Res) was prepared by polyethylene glycol (PEG) modification, which provided theoretical guidance for material design.
• Nisin and Res have synergistic inhibitory effects on methicillin-resistant *S. aureus*.
• α-hemolysin can break down liposomes and trigger drug release, laying a theoretical foundation for the development of materials targeting methicillin-resistant S. aureus enteritis.

## Graphical abstract

**Figure:**
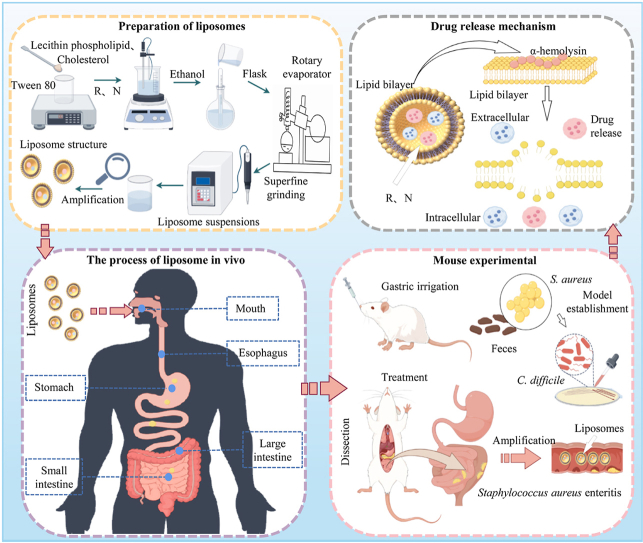

## 1 Introduction

In the post-antibiotic era, the treatment of bacterial infections mainly depends on antibiotics. However, antibiotic resistance has become a global health crisis [1]. According to surveys, in the United States alone, more than 2 million people are infected with antibiotic-resistant bacteria annually, and at least 23,000 people die from complications related to antibiotic-resistant infections. According to estimates, the number of deaths from multidrug-resistant bacteria will exceed the number of deaths from cancer by 2050 [2]. Antibiotic-associated diarrhea (AAD) is a common multifactorial gastrointestinal complication during anti-infective therapy. However, numerous investigations have demonstrated that the primary bacteria responsible for AAD include *Klebsiella oxytoca*, *Clostridium difficile*, *Clostridium perfringens*, and methicillin-resistant *S*. *aureus* (MRSA). Antibiotic-associated enterocolitis (AAE) caused by MRSA is a more serious manifestation of AAD [3]. MRSA is the most common hospital-acquired resistant bacteria. MRSA usually overgrows in the intestine to alter the composition of the intestinal microbiota and produce a variety of toxins, including the superantigen staphylococcal enterotoxins, which damage surrounding tissues and eventually lead to diarrhea and intestinal inflammation in patients [4]. The primary treatment approach for AAE at the moment involves the use of antibiotics like linezolid, metronidazole, and vancomycin. However, excessive and prolonged use of antibiotics causes microbial resistance as well as treatment inefficiency because they are not targeted. Therefore, there is an urgent need to develop novel antibacterial therapies targeting this pathogen to address the challenge.

The research of antibacterial medications with novel targeting or mechanisms of action has emerged as a new pharmacological strategy with a continuous search and exploration of new antibacterial agents. Natural antimicrobial agents extracted from natural materials or their by-products represent an innovative and sustainable approach to combating foodborne pathogens and human clinical diseases. Nisin is a cationic antimicrobial peptide produced by *Lactococcus lactis* that has been shown in numerous studies to have significant inhibitory effects on a variety of pathogens, including *Listeria monocytogenes* and *S*. *aureus* [5]. At the same time, nisin (Nisin) is also the only bacteriocin widely used in commercial applications, serving as a food additive in various fields such as eggs, dairy products, meat, and fish [6]. Nonetheless, nisin has remarkable stability at pH 3, with less than 5 % activity loss even in high-pressure sterilizing conditions. However, its instability occurs at room temperature as soon as the pH value surpasses 6 [7]. Resveratrol (Res), as a natural non-flavonoid polyphenolic substance mainly found in plants such as grapes, peanuts, and Polygonum cuspidatum, offers several health benefits, including anti-aging, anti-cancer, anti-inflammatory, anti-oxidation and prevention of cardiovascular diseases [8]. The broad therapeutic potential and safety of Res treatment make it an attractive candidate for developing new pharmaceutical products [9]. Res can have certain drawbacks when used, though, including poor water solubility, insufficient stability caused by photoinduced isomerization, and autoxidation [10]. The unstable properties of Res and Nisin limit their utilization, which greatly hinders their potential for application in drug delivery [11]. Therefore, improving the stability and bioavailability of Res and Nisin is critical to expanding their applications in the food and pharmaceutical industries.

With the rapid development of nanotechnology, the application of nanomaterials has attracted more and more attention. As a nano-drug carrier, liposomes have become the most explored and widely used drug carrier in targeted drug delivery systems [12]. It can encapsulate hydrophilic or lipophilic drugs by using the affinity of different parts of the closed phospholipid bilayer vesicles. Furthermore, because liposomes as carriers have a structure similar to cell membranes, they not only exhibit good biocompatibility and immunogenicity but also have the advantages of protecting bioactive components, prolonging drug half-life, reducing toxicity, and increasing efficiency [13].

**Fig. 1:**
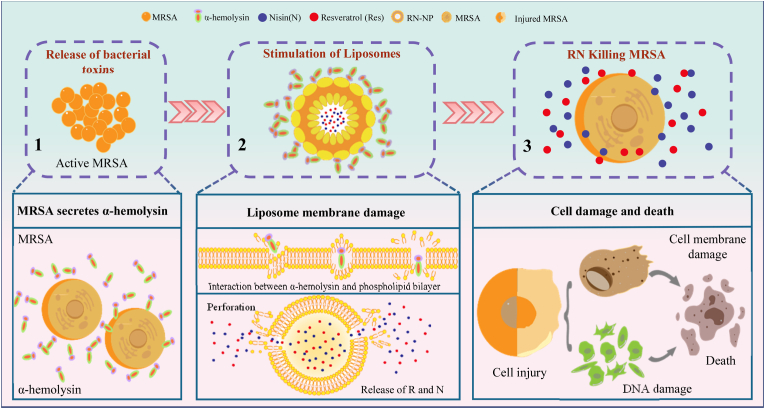
A schematic diagram of RN release from liposomes induced by α-hemolysin secreted by MRSA.

*S*. *aureus* can secrete α-hemolysin, which is the prototype of a small β-barreled pore-forming cell toxin. Recent studies have reported that the pore-forming toxin (PFT) secreted by bacteria can pierce the cell membranes [14]. Liposomes are spherical lipid vesicles with a double-layer membrane structure composed of amphiphilic lipid molecules, similar to cell membranes. As far as we know, there are few reports on the interaction between PFT and liposomes. Therefore, combining the characteristics of PFTs secreted by bacteria and liposomes may provide a new and safer controlled release mechanism. Specifically, PFTs secreted by bacteria form pores in the liposomes, there triggering the release of bioactive substances from within. Given the above situation, inspired by the effect of pore-forming toxins on cell membrane damage, we have developed a nano-carrier system with good stability for targeted treatment of MRSA enteritis. As shown in Fig. 1, this is a passive selection strategy. The α-hemolysin secreted by MRSA triggers the release of antimicrobial agents (RN) from liposomes by destroying spherical lipids with a bilayer membrane structure. In other words, the liposome-encapsulated antibacterial agent will not be released until the liposomes encounter an infectious target bacteria that secretes α-hemolysin. The released RN will then rapidly and locally exert its antibacterial activity. As a proof of concept, we demonstrated here that PFT can be used to trigger the release of RN from liposomes, and the released RN can effectively kill MRSA that secretes PFT. In addition, the efficacy of liposome-encapsulated RN in the treatment of MRSA enteritis was also evaluated.

## 2 Experiment and methods

### 2.1 Materials

Methicillin-resistant *S*. *aureus* ATCC 33591 and *E. coli* CCTCC AB93154 were purchased from the China Center for Type Culture Collection (CCTCC, Wuhan, China). Resveratrol (purity >99 %), nisin type A (≥1000UI/mg), soybean lecithin (purity >98 %), cholesterol (purity >98 %), Tween 80, and other reagents were purchased from Shanghai McLean Biochemical Technology Co., Ltd. J774A.1 mouse macrophages were obtained from the National Certification Cell Culture Collection (Shanghai, China).

SPF male Kunming mice (5–6 weeks) were purchased from Jinan Pengyue Experimental Animal Breeding Co., Ltd. (License No.: SCXK 2022 0006). Mice were fed a standard rodent diet, free to drink water, and caged at 22–25 °C. The animals and programs of this study have been approved by the Animal Care and Use Committee of Qingdao Agricultural University, and follow the national experimental animal ethics guidelines.

### 2.2 Determination of cell membrane integrity

MRSA suspension (10^6^ CFU/mL) was incubated with 1 MIC Res, 1 MIC Nisin, or a mixture of both for 5–10 min. Afterward, the bacterial cells after centrifugation at 5000 rpm were resuspended in 400 μL PBS (0.01 M, pH 7.2) and incubated with 4 μL propidium iodide (PI) in the dark at 25 °C for 30 min. Finally, high-speed flow cytometry (BD FACS Aria III) was used for detection [11].

### 2.3 DNA of MRSA

The effect of different antibacterial agents on the genomic DNA of MRSA was studied by gel retardation assay [15]. The genomic DNA of MRSA was extracted using a centrifugal column bacterial genomic DNA extraction kit (Beijing Tiangen Biochemical Technology Co., Ltd.). The optical density at 260 nm and 280 nm was measured by an ultraviolet spectrophotometer to evaluate whether the DNA concentration reached the experimental concentration. The extracted DNA (50 μg/mL) was treated with the antibacterial agent at 37 °C for 1 h and 10 μL of the sample was mixed with 6 × 2 μL of DNA loading buffer. The mixture was analyzed by 1 % agarose gel electrophoresis.

### 2.4 Observation of cell surface morphology of MRSA

The surface morphology of MRSA cells was observed by scanning electron microscopy (SEM) [16]. The MRSA cell suspension (2 × 10^8^ CFU/mL) was incubated with the antibacterial agent at 37 °C for 1h, and the collected cells were washed with PBS (0.01 M, pH 7.2). Then, the cells were fixed overnight with 2.5 % glutaraldehyde at 4 °C. After washing with PBS, the solution was dehydrated in a gradient manner with ethanol solution and replaced twice with isoamyl acetate. Finally, the cells were fixed on the SEM scaffold, vacuum-sputtered gold, and observed.

### 2.5 Preparation and modification of nanoliposomes

Res and Nisin nanoliposomes (RN-NPs) were prepared by the film-ultrasonic dispersion method [17]. A certain amount of soybean lecithin, cholesterol, and Tween 80 (6:1:1.8, w/w) were mixed and dissolved in anhydrous ethanol. After stirring to be completely dissolved, a rotary evaporator was used to remove anhydrous ethanol by heating it in a 60 °C water bath to form a lipid film. A certain amount of Res and Nisin solution was added, and phosphate buffer (PBS, 0.03 M, pH 7.2) was added to rehydrate the film. The film was completely dissolved to form a uniform milky white solution so that the lipid concentration was 28 mg/mL. Nanoliposomes were obtained by using a cell ultramicro pulverizer, working for 10 s, intervals of 5 s, and power of 300 W for 30 min. Subsequently, an equal volume of phosphatidylethanolamine-polyethylene glycol 2000 (DSPE-PEG2000) aqueous solution with a mass fraction of 3 % was added to the prepared nanoliposome solution, and it was uniformly mixed using a vortex. The solution was allowed to stand at 4 °C for 1 h and filtered with a 0.22 mm microporous membrane to obtain polyethylene glycol-modified nanoliposomes (RN-NPs).

### 2.6 Size and zeta potential of nanoliposomes

Referring to Liu et al.’s method, the average particle size, PDI, and potential of liposomes were measured by the Malvern laser particle size analyzer [18]. At 20 °C, the refractive index of phospholipids (1.490) relative to the refractive index of the dispersion medium (1.330) is 1.120, and the absorption rate of phospholipids is 0.001. All samples were diluted 10 times with ultrapure water, and each sample was measured in parallel more than three times.

### 2.7 Encapsulation of antibacterial drugs

The standard solution of Res and Nisin was prepared and the standard curve was drawn [19]. Liposomes were separated by a 30 kDa ultrafiltration tube and filtered using a 0.22 μm membrane. Add the same amount of ethanol demulsifier, ultrasonic treatment at 25 °C for 1 h, standing for 24 h, filtration, using C18 column high-performance liquid chromatography to determine the total concentration of Res and Nisin in the filtrate. The content and encapsulation efficiency of Res and Nisin in the samples were calculated according to the peak area and standard curve.

### 2.8 Microstructure

The morphology of liposomes was observed by laser scanning confocal microscope (CLSM). 40 μL of Nile red dye (0.25 mg/mL) was added to 500 μL liposome samples and mixed evenly. Drop 10 μL of sample onto a confocal microscope slide, half dry at room temperature, and cover with a cover glass. First, a low-power microscope is used to find the appropriate field of view, and then a high-power lens is used for observation.

### 2.9 Determination by fourier transform infrared (FTIR)

FTIR spectrometer was used to determine the encapsulation of antimicrobial substances by liposomes [18]. The liposomes were freeze-dried. Then, Res, Nisin, blank liposome, and RN-NPs were scanned to obtain FTIR spectra: MCT-A infrared detector was used with a resolution of 8 cm^−1^. The infrared spectrum scanning range was 4000–500 cm^−1^, and each image was scanned 64 times.

### 2.10 Investigation into stability

RN-NPs were stored at 4 °C, 22 °C and 37 °C for 7 days, respectively. The average particle size, PDI, and Zeta potential of liposomes were measured at different time intervals (1 day) to determine the stability of liposomes.

### 2.11 *In vitro* experiments

Sodium chloride (NaCl), hydrochloric acid (HCl), dipotassium hydrogen phosphate (K_2_HPO_4_), and sodium hydroxide (NaOH) were used to configure simulated gastric fluid (SGF) and simulated intestinal fluid (SIF). The pH of SGF was adjusted to 1.2 with 0.1 M HCl solution, and the pH of SIF was adjusted to 7.4 with 0.01 M NaOH solution. Nanoliposomes were mixed with SGF and SIF solution at 1:3 (v/v), and pepsin and trypsin were added to make the concentration of 3.2 mg/mL, 37 °C constant temperature water bath, 95 rpm shake evenly. Sampling analysis was performed at certain digestion times.

### 2.12 Determination of microstructure and lipolysis reaction

In the digestion experiment, the liposome with a certain digestion time (0 h, 2 h, 4 h) was used to observe the surface morphology by optical microscope. The lipolysis reaction of liposomes in vitro was determined by monitoring pH and automatic acid-base titration. The liposomes were mixed with SIF solution and the pH was adjusted to 7.4. Adding 3.2 mg/mL trypsin and bile salt and stirring at a constant temperature of 37 °C water bath, the pH value was adjusted to 7.40 ± 0.01 with 0.01 M NaOH. Under the same conditions, the standard curve of free fatty acid release of oleic acid was drawn.

### 2.13 Study on the mechanism of pore-forming toxin stimulation release *in vitro*

Phosphate buffer (PBS) was used to adjust the concentration of *E. coli* and MRSA to 10^6^ CFU/mL, and RN-NPs were added to the bacterial test tube. To detect the release of α-hemolysin in MRSA, we conducted a hemolysis assay. The prepared MRSA suspension was centrifuged at 10,000 rpm for 2 min at 4 °C, and the supernatant was collected. Then, 25 μL of sheep red blood cells (5 × 10^6^ cells/mL) and 75 μL of the supernatant were added to 900 μL of PBS buffer and incubated at 37 °C for 15 min. The mixture was then centrifuged at 6000 rpm for 1 min at room temperature. The collected supernatant was measured for OD at 543 nm to determine the concentration of α-hemolysin [20]. It was cultured at 37 °C, 150 rpm, and the number of residual bacteria in different groups was measured at different time points. At the same time, the contents of Res and Nisin in the samples incubated for 24 h were analyzed by high-performance liquid chromatography.

### 2.14 Study on cell viability

The cytotoxicity of nanoparticles was determined by MTT assay [21]. Mouse macrophages J774A.1 were incubated in 96-well plates at 37 °C and 5 % CO_2_ overnight. Cells were washed with Hanks buffer (HNSS) preheated to 37 °C. While setting the blank group, 100 μL nanoparticles with different concentration gradients (0.01–5 mg/mL) were added to each well and incubated at 37 °C for 4 h. Subsequently, 0.5 mg/mL MTT solution was added to each well and incubated at 37 °C for 3 h. After removing the excess MTT solution, 200 μL dimethyl sulfoxide (DMSO) was added to form a blue-purple crystal. The absorbance at 560 nm was measured by a microplate reader and the cell viability was calculated.

### 2.15 Hemocytolysis

The hemolysis test of RN-NPs was performed using fresh mouse blood [22]. Since the international hemolysis standard is 5 %, we use this as an evaluation standard for experiments. The absorbance of different media was measured at 570 nm with PBS as negative control and water as positive control. The calculation formula is as follows:

$$RHR\;\left(\%\right)=\frac{A_{\text{sample}}-A_{PBS}}{A_{water}-A_{PBS}}\times 100\%$$

### 2.16 Study on anti-inflammatory activity *in vitro*

J774A.1 cells were used to detect the anti-inflammatory activity of nanoparticles *in vitro*. 10^5^ cells per well were inoculated into 48-well plates and incubated overnight. The cells were rinsed with HBSS solution preheated at 37 °C, and 300 μL of lipopolysaccharide (LPS) at a concentration of 100 ng/mL was added to each well for incubation for 20 h. After incubation, 30 μL of nanoparticles with different concentrations were added and incubated for 4 h. Among them, RN-NPs were broken to release the RN. The supernatant of each well was collected and centrifuged to remove cell debris. The content of TNF-α was determined by the ELISA (Invitrogen, USA) quantitative kit, and the total protein content was determined by the BCA protein detection kit. Flow cytometry was used to assess the ROS scavenging and anti-apoptotic activity of RN-NPs. First, viable cells were pretreated, and J774A.1 cells were exposed to 500 μM hydrogen peroxide (H_2_O_2_) for 1 h. They were then cultured with R, N, RN or RN-NPs for 24 h. Finally, the cells were processed into a suspension, stained with Hoechst 33342 and PI, and collected for flow cytometry analysis.

### 2.17 Construction of MRSA enteritis model

To ensure that the antibiotic mixture used in the experiment effectively induces intestinal microbiota imbalance and provides suitable conditions for the subsequent colitis induction experiment while minimizing toxicity to the mice, we optimized the drug types and dosages based on previous animal experiment data and relevant literature [3,23]. A mixture of antibiotics consisting of kanamycin (0.6 mg/mL), gentamicin (0.0525 mg/mL), colistin sulfate (1275 U/mg), metronidazole (0.3225 mg/mL), vancomycin (0.0675 mg/mL) and cefaclor (0.45 mg/mL) was added to the drinking water of mice. After 4 days of pretreatment, the mice were inoculated with 10^7^ CFU of MRSA after drinking normal drinking water for 1 day [3]. On the 11th day, mice were given free RN or RN-NPs or positive drug oxacillin sodium by intragastric administration. The administration time lasted for 6 days, and the mice were sacrificed on the 18th day.

### 2.18 Body weight and clinical activity score of mice

Each mouse in each group was scored for clinical activity. Weight loss, fecal consistency, and rectal bleeding were used to calculate the score (0–4).

### 2.19 Myeloperoxidase (MPO) activity

The mice were quickly frozen in liquid nitrogen and kept at −80 °C immediately after the intestine was dissected. Accurately weigh the weight of the animal tissues. According to the ratio of weight (mg): volume (μL) = 1:9, 9 times the volume of homogenate medium (0.9 % normal saline) was added. Under the condition of the ice water bath, the mechanical homogenate was prepared into 10 % homogenate, 2500–3000 r/min, centrifuged for 10 min, and the supernatant was taken for determination. MPO kit (A044-1, Nanjing Jiancheng) was used to determine each group. 60 °C water bath for 10 min, then immediately at 460 nm wavelength, 1 cm light path, distilled water zero, read the absorbance [24].

### 2.20 Quantification of tissue cytokines

After collecting the supernatant of intestinal tissue of experimental mice, the contents of IL-6 and IFN-γ were determined by Mouse IL-6 ELISA Kit and Mouse IFN-γ ELISA Kit (Invitrogen, USA) [25]. After washing, sample addition, incubation, antibody addition, enzyme conjugate addition, and chromogenic substrate addition, 100 μl of 2 M sulfuric acid was added to each reaction well to terminate the reaction. Within 10 min, the OD value of each hole was measured by using the microplate reader at the detection wavelength of 450 nm after zeroing the blank control hole. Finally, the standard curve was made according to the concentration and OD value of the standard, and then the sample concentration was calculated according to the standard curve equation.

### 2.21 Histological evaluation

The samples were fixed with 4 % paraformaldehyde solution overnight (12 h), and histological evaluation was performed after the fixation was in good condition. The tissue sections were fixed by paraffin embedding. Two groups of three 10 μm slices were cut at an interval of 150 μm. The sections were stained with hematoxylin-eosin (H&E). The histological damage score was evaluated by blind method, and the histological damage score was expressed as a percentage of total tissue damage [26].

### 2.22 Data and statistical analysis

The experiment was repeated at least 3 times, and all data were expressed as mean ± standard deviation. The Tukey multiple comparison test of GraphPad software was used for one-way analysis of variance to evaluate the differences between groups further. P < 0.05 was considered statistically significant (∗p < 0.05, ∗∗p < 0.01, ∗∗∗p < 0.005, ∗∗∗∗p < 0.001), while P > 0.05 was not statistically significant (ns).

## 3 Results and discussion

### 3.1 The synergistic antibacterial mechanism of Res and Nisin

**Fig. 2:**
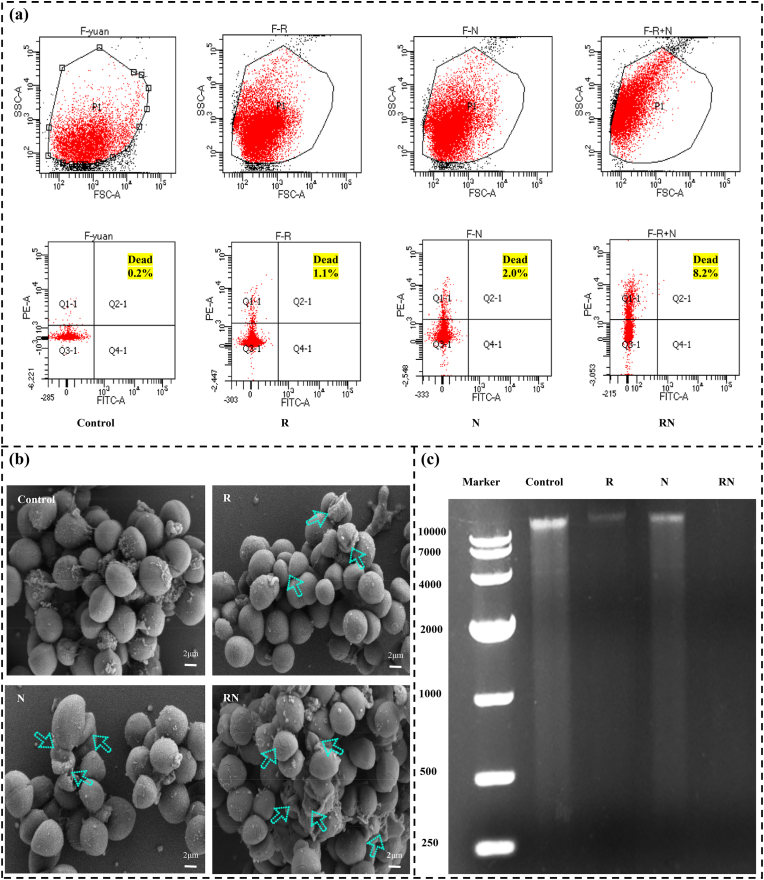
Flow cytometry results (A), SEM images (B), and genomic DNA changes (C) of MRSA incubated with control, Res or Nisin alone, and combination therapy.

In the previous study, it was found that the combination of Res and Nisin (RN) had a synergistic inhibitory effect on MRSA, but the specific synergistic antibacterial mechanism was still unclear. Consequently, the detrimental effect of the RN combination on the MRSA cell membrane was examined using flow cytometry to better understand the synergistic antibacterial mechanism of RN against MRSA. The results showed that the mortality rate of MRSA cells in the control group was only 0.1 %, which could be attributed to normal cell death caused by cell senescence or some mechanical damage during processing. On the other hand, the cell death rates of MRSA in the Res, Nisin, and RN treatment groups were 1.1 %, 2.0 %, and 8.2 %, respectively (Fig. 2a). It could be seen that compared with the single Res and Nisin treatment group, the cell mortality of MRSA in the RN combined treatment group increased by 7.45 times and 4.1 times, respectively. To confirm the synergistic impact of RN, additional research was conducted to examine the consequences of RN treatment on the morphology of MRSA (Fig. 2b). The results show that compared to the control group, the MRSA in the RN combination treatment group exhibited cell shrinkage, rupture, and leakage of intracellular contents, indicating severe damage to cell integrity. In contrast, only mild shrinkage was observed in the MRSA cells treated with either Res or Nisin alone. It is obvious that the RN combination had a significant detrimental impact on the MRSA cell membrane. For additional proof, the combination of RN and therapy damaged MRSA's cell membrane and caused cell contents to degrade or leak out. Through preliminary literature review and experimental studies, we hypothesize that the DNA damage of MRSA caused by Res is primarily achieved through the following mechanisms: inhibition of DNA synthesis, induction of DNA damage and oxidative stress, and suppression of bacterial virulence gene expression [[27], [28], [29]]. Meanwhile, Nisin can disrupt the cell membrane and inhibit cell wall synthesis, thereby facilitating the entry of Res into the bacterial cells, allowing it to interact with DNA and accelerate its damage and degradation. Additionally, nisin may also promote the accumulation of reactive oxygen species (ROS), leading to further oxidative damage to DNA [30]. In the study conducted by Liu et al., qPCR was used to detect gene expression changes in *S. aureus* JE2 strains after Res treatment. The results indicated that Res treatment did not affect the expression of *recJ*, *xerC*, and *xseA* genes but significantly upregulated the expression of *lexA* and *recA*, which are related to the SOS response [31]. This finding further supports the notion that Res induces DNA damage in bacteria. Therefore, the changes in the genomic DNA of MRSA were analyzed by gel electrophoresis (Fig. 2c). The DNA bands in the control group showed bright fluorescence. The fluorescence intensity of DNA bands in the Res or Nisin group alone was significantly weaker than that in the control group, especially the DNA bands in the RN combined treatment group had almost no fluorescence, indicating that the DNA of MRSA was almost completely degraded.

Previous studies have also confirmed that Nisin can destroy Gram-positive bacteria by forming pores on the cell membrane and binding to lipid II to inhibit the biosynthesis of cell walls [32]. Likewise, Res has also been shown to have a significant inhibitory effect on MRSA. PI staining and SEM showed that Res could destroy the integrity of the cell membrane [33]. Furthermore, Res can also increase the expression of stress (SOS) response genes *LexA* and *RecA*, thereby interfering with and damaging the integrity of DNA in *S*. *aureus* [34]. It can be seen that the main mechanisms by which Nisin and Res exert antibacterial effects are related to the destruction of cell membrane integrity. Therefore, when the two are combined, they accelerate the damage to the cell membrane.

### 3.2 Construction and characterization of nanoliposomes

**Fig. 3:**
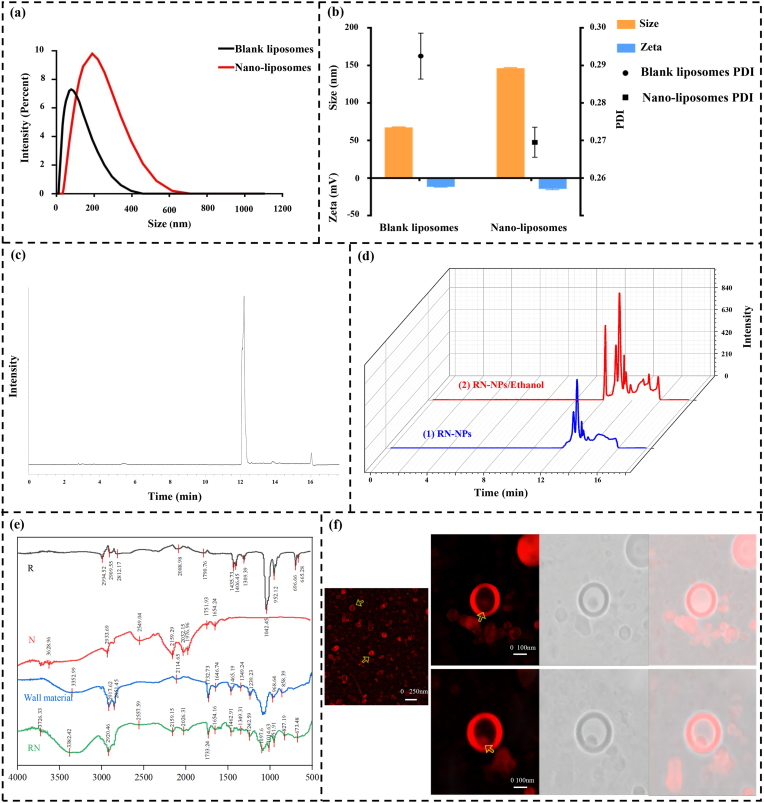
Characteristics of nanoliposomes. (A) The average particle size and frequency distribution (B), PDI and Zeta of blank liposomes and RN-NPs; (C) High-performance liquid chromatography of Res and Nisin standard; (D) The blue chromatogram was RN-NPs, and the red chromatogram was the high-performance liquid chromatogram of RN-NPs after incubation with ethanol. (E) FTIR images of Res, Nisin, wall material, and RN-NPs; (F) CLSM images of RN-NPs.

The particle size, Zeta potential, and polydispersity index (PDI) of RN-NPs are shown in Fig. 3a and b. The average particle sizes of blank nanoliposomes and RN-NPs were 67.67 ± 0.53 nm and 146.50 ± 0.83 nm, respectively. It can be seen that the average particle size of RN-NPs was slightly larger than that of blank nanoliposomes. The Zeta potential of blank nanoliposomes was −11.87 ± 0.22 mV, while the Zeta potential of RN-NPs was −14.61 ± 1.03 mV. The larger the absolute value of Zeta potential indicates that the stronger the charge on the surface of the particles, the stronger the repulsion between the particles. According to the above research, RN-NPs have a larger repulsive force than other NPs, meaning they can effectively prevent liposome aggregation and increase system stability. In addition, it can be seen from Fig. 3b that the PDI values of blank nanoparticles and RN-NPs were less than 0.3, indicating that the liposomes were uniformly dispersed and stable.

The encapsulation efficiency of Res and Nisin in RN-NPs was determined using High-Performance Liquid Chromatography (HPLC), and a control experiment was conducted to verify whether RN was successfully embedded. Firstly, the standard samples of Res and Nisin were analyzed by HPLC, and the retention times of the characteristic peaks of Res and Nisin in the chromatogram were determined to be 12.20 min and 16.03 min, respectively (Fig. 3c). Secondly, HPLC was used to analyze the untreated RN-NPs solution, and the chromatogram showed no characteristic peaks for Res and Nisin, indicating that no free Res or Nisin existed in the RN-NPs solution (Fig. 3d, blue chromatogram). To verify whether RN was successfully encapsulated in liposomes, we incubated the RN-NPs solution with an equal amount of ethanol demulsifier to promote the rupture of the liposome membranes, thereby releasing Res and Nisin. Subsequently, the treated ethanol/RN-NPs samples were subjected to HPLC detection again (Fig. 3d, red chromatogram). Compared with the untreated RN-NPs control group, the chromatogram of the ethanol/RN-NPs group showed characteristic peaks of Res and Nisin at 12.21 min and 15.89 min, respectively, and the peak areas of Res and Nisin increased significantly. This result indicated that RN was successfully encapsulated in liposomes and released after the liposome membrane was destroyed. Finally, based on the change in peak areas before and after the rupture of RN-NPs and the standard curve, the embedding rate of Res was 35.45 %, and that of Nisin was 29.10 %. In conclusion, this experiment successfully verified the encapsulation of Res and Nisin in RN-NPs, which provided basic data support for the stability and effectiveness of RN-NPs in practical applications.

The RN-NPs were further characterized by fourier transform spectroscopy (FTIR) and laser confocal microscopy (CLSM) (Fig. 3e and f). According to the data, Res had an obvious hydroxyl absorption peak between 2917 and 2995 cm^−1^. The obvious ester (C=O and C-O) absorption peaks were observed at 1790.76 cm^−1^ and 1435.73 cm^−1^, respectively [35]. Nisin observed the axial stretching of O-H and N-H at 3628.96 cm^−1^. The asymmetric stretching vibration of -NH^3+^ was observed at 2933.69 cm^−1^, and its specific vibration frequency was related to the amide B band. Around 1654.24 cm^−1^, a stretching vibration corresponding to the C=O bond of amide I was observed [36]. The lecithin in the wall material showed stretching vibrations of COO- in C=O at 1732.73 cm^−1^. RN-NPs had a strong absorption peak at 3382.42 cm^−1^, which was related to the N-H stretching of Nisin and the stretching vibration of lecithin, indicating that the formation of liposomes caused the formation of hydrogen bonds between lecithin and Res or Nisin, thereby shifting the absorption peak to the left. The absorption peak of RN-NPs at 2920.46 cm^−1^ proved that the stretching vibration of C-H in Nisin shifted to the low wavenumber direction due to binding [37]. At the same time, the characteristic peaks of Res were found at 2920.46 cm^−1^ and 1733.24 cm^−1^. In conclusion, FTIR analysis showed that Res, Nisin, and lecithin primarily interacted through hydrogen bonding and electrostatic interactions. Finally, CLSM was used to view the liposomes' microstructure. From Fig. 3f, it can be seen that the prepared nanoliposomes exhibit a spherical or near-spherical vesicle structure, with a clearly distinguishable outer bilayer phospholipid membrane and an internal aqueous phase. The high resolution of confocal microscopy allows for a more detailed visualization of the internal structure of the liposomes. In short, the results showed that RN-NPs particles not only had smooth surfaces and regular spherical shapes but also exhibited good dispersion and no agglomeration. At the same time, the bioactive components embedded in the liposome can also be clearly seen through the image.

### 3.3 *In vitro* stability and digestion simulation

**Fig. 4:**
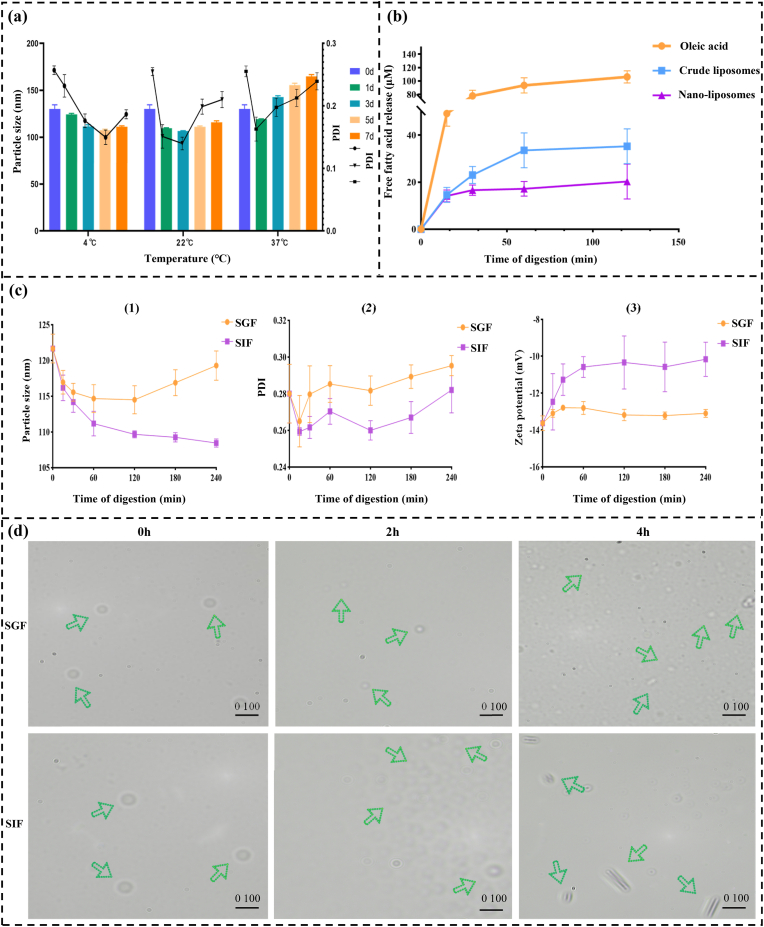
(A) The changes of average particle size, PDI, and Zeta potential of RN-NPs at 4 °C, 22 °C and 37 °C; (B) Hydrolysis stability in simulated intestinal fluid; (C) The changes of average particle size (1), PDI (2) and Zeta potential (3) of RN-NPs during SGF and SIF digestion. (D) Microstructure changes of the coarse RN-NPs during SGF and SIF digestion.

After the characterization of RN-NPs, the stability of liposomes was further analyzed. As shown in (Fig. 4a), RN-NPs were stored at different temperatures (4 °C, 22 °C, and 37 °C) for 7 days. The results showed that the average particle size and PDI of RN-NPs initially decreased and then increased. The observed phenomenon may be attributed to changes occurring at different storage stages. In the early phase, the weakening of hydrogen bonding and ester bonding capacity in liposomes leads to a reduction in average particle size. In the later phase, the electrostatic interactions between liposomes diminish, causing some liposomes to aggregate, which results in an increase in both average particle size and PDI. Previous studies have also confirmed that the oxidation of lipid components within liposomes or hydrolysis of ester bonds that hold lipid components together can contribute to liposomal degradation [38]. Additionally, research by Santhosh et al. suggests that when charged lipids are used in liposome preparation, the electrostatic repulsion enhances liposomal stability, preventing aggregation and fusion of adjacent vesicles. Conversely, when surface charge decreases, the stability of phosphatidylcholine liposomes is significantly reduced [39]. Compared with RN-NPs stored at 4 °C, the average particle size of RN-NPs stored at 22 °C and 37 °C changed more obviously. This is because molecular thermal motion is influenced by temperature. In the later stage of storage, the increased temperature will accelerate the thermal movement of the particles, resulting in a decrease in electrostatic repulsion, thereby promoting the collision and aggregation between liposomes. It is evident that RN-NPs stored at 4 °C exhibited the smallest change in average particle size and had the best stability. At 22 °C and 37 °C, RN-NPs do not undergo significant changes within a short period (2 days). Therefore, it is recommended to store RN-NPs in a low-temperature environment before human application and to minimize their exposure to room temperature to reduce any impact on liposome stability.

Then, to evaluate the stability of nanoliposomes in simulated intestinal fluid (SIF), the hydrolysis behavior and stability of liposomes during digestion were investigated by measuring the release of free fatty acids from crude liposomes and nanoliposomes in SIF. Firstly, the amount of free fatty acid released at a known concentration of oleic acid (0–15000 μmol) was used as a measure of hydrolysis. The degree of hydrolysis of crude liposomes and nanoliposomes was compared by measuring the release of free fatty acids during digestion. The quantity of free fatty acids released in SIF by crude liposomes and nanoliposomes is significantly less than that of oleic acid, as (Fig. 4b) illustrates. This indicates that the structure of liposomes can prevent or delay the release of free fatty acids to a certain extent. Furthermore, compared with nanoliposomes, crude liposomes released more free fatty acids during the whole digestion process, indicating that the hydrolysis rate of crude liposomes in SIF was higher. Crude liposomes' low surface area to volume ratio and potential for defects or irregularities in the liposome membrane structure could be the primary causes of this behavior. This physical structural instability makes crude liposomes more prone to deformation, aggregation, or fusion, thus increasing the release of free fatty acids. In contrast, nanoliposomes possess a more compact and stable structure, providing stronger resistance to the hydrolysis reaction in SIF. Additionally, it is worth noting that the free fatty acid release of nanoliposomes began to stabilize at the early stage of digestion, while the crude liposomes reached a steady state after a long time. This phenomenon may be due to the fact that the modification of PEG on the surface of nanoliposomes resists the damage of SIF to the liposome layer to a certain extent. In summary, these results showed that nanoliposomes exhibited higher stability in SIF, releasing significantly fewer free fatty acids than crude liposomes and showing a lower hydrolysis rate from the early stages of digestion. This stability can be attributed to the tightness and uniformity of the nanoliposome structure, which can effectively resist the influence of the hydrolysis reaction during the digestion process. Therefore, nanoliposomes can provide better protection for the application of drugs in the gastrointestinal tract.

To ensure that RN-NPs can resist gastric digestion and successfully reach the affected area in the intestines, we continued to analyze the changes in RN-NPs during an *in vitro* simulated gastrointestinal digestion process (Fig. 4c). The changes in the average particle size and PDI of RN-NPs are shown in (Fig. 4c1 and Fig. 4 c2). The average particle size of RN-NPs showed minimal change during the simulated gastric fluid (SGF) digestion phase, indicating that the liposomal membrane of RN-NPs can effectively resist the influence of low pH and pepsin so that RN-NPs can pass through the stomach and reach the intestines smoothly. After entering the simulated intestinal fluid (SIF) digestion stage, the average particle size of RN-NPs began to decrease and showed a continuous downward trend. The reason for this phenomenon is mainly due to the emulsification of bile salt on RN-NPs in SIF and the hydrolysis of liposome membrane by trypsin. Nevertheless, even at the end of the SIF digestion process, the average particle size of RN-NPs remained larger than that of blank nanoliposomes, indicating that RN-NPs still maintained a certain level of structural integrity in the intestinal environment. At the same time, the liposomes effectively protected the stability of RN, preventing it from being prematurely absorbed or degraded [40]. Additionally, as shown in (Fig. 4c2), the PDI values of RN-NPs remained stable throughout the digestion phase, demonstrating their good dispersibility and uniformity. This indicates that RN-NPs can maintain a stable dispersion state in the simulated gastrointestinal environment and are not prone to significant aggregation or precipitation. Moreover, the results of (Fig. 4c3) showed that the Zeta potential of RN-NPs remained relatively stable in SGF, while the absolute value of Zeta potential decreased significantly when it was digested by SIF. This suggests that the strongly acidic environment of SGF has little effect on the surface charge of the liposomal membrane, whereas the presence of anions such as bile salts in SIF leads to a decrease in the surface charge of the liposomal membrane [41]. Since the coarse RN-NPs share the same structure as RN-NPs but have a larger volume, making their external changes more observable during microscopic structural analysis, they were selected as a substitute for RN-NPs in subsequent experiments. Optical microscopy was employed to investigate the morphological changes of RN-NPs in simulated gastrointestinal fluids to more clearly demonstrate those changes (Fig. 4d). During the digestion of SGF, the coarse RN-NPs maintained a regular spherical structure without significant morphological changes, which further proved its stability in gastric juice. In the early stage of SIF digestion, the morphology of the coarse RN-NPs remained basically unchanged, but the liposome membrane showed slight swelling and thickening. With the progress of the digestion process, especially in the later stage of digestion, although some coarse RN-NPs began to aggregate and began to appear irregular flake or strip shape, the coarse RN-NPs were still not degraded.

In summary, through the changes of average particle size, PDI, Zeta potential, and microstructure of RN-NPs during simulated gastrointestinal digestion, it was found that RN-NPs had good stability in gastric juice and could resist the effects of gastric acid and pepsin, thus successfully reaching the intestine. After reaching the intestine, although RN-NPs were subjected to bile salt emulsification and trypsin hydrolysis, some of the RN-NPs were changed in shape, but they were still not degraded. The primary cause of this phenomenon is due to the modification effect of polyethylene glycol (PEG). It can be seen that RN-NPs have good stability in the gastrointestinal tract, which provides a guarantee for its targeted release at the inflammatory site. Previous studies have shown that PEGylated liposomes can improve the dispersibility of liposomes by site blocking, and increase the repulsive force between liposomes and serum components, to effectively make up for the limitations of liposomes in enhancing the permeability and retention effect (EPR), thus prolonging the retention time of liposomes *in vivo* and enhancing the therapeutic effect of drugs [[42], [43], [44]]. Therefore, we selected phosphatidylethanolamine-polyethylene glycol 2000 (DSPE-PEG2000), a PEG derivative with both phospholipid amphiphilicity and high hydrophilicity, to modify RN-NPs. This modification functionalizes the surface of RN-NPs, achieving an “invisible” state that reduces the impact of the reticuloendothelial system (RES) on RN-NPs, prolonging their retention time at the inflamed site in the intestine and enhancing therapeutic efficacy [36]. The specific mechanism of action is as follows: First, α-hemolysin, as a multi-subunit protein, typically exists in an inactive monomeric form dissolved in liquid. Upon contact with the target cell membrane, it undergoes a conformational change, transitioning into a more active polymeric form. This structural transformation enables α-hemolysin to form effective membrane-penetrating pores on the cell membrane [20]. Subsequently, the N-terminus of α-hemolysin exhibits lipophilicity, allowing it to embed into the lipid bilayer of the liposomal membrane. Through interactions with specific phospholipid molecules on the membrane, it induces membrane perforation. These pores compromise membrane integrity, increasing permeability and facilitating the release of drug molecules encapsulated within the liposomes. Finally, PEG modification effectively helps evade immune system clearance, prolonging circulation time in the body. Under the protective effect of PEG, RN-NPs can successfully reach regions with higher α-hemolysin concentrations, achieving precisely targeted release. In summary, RN-NPs can successfully reach the intestine without being affected by gastric fluids while enhancing drug concentration at the lesion site and minimizing side effects on healthy tissues. This significantly improves the precision and effectiveness of the treatment.

### 3.4 Releasing mechanism

**Fig. 5:**
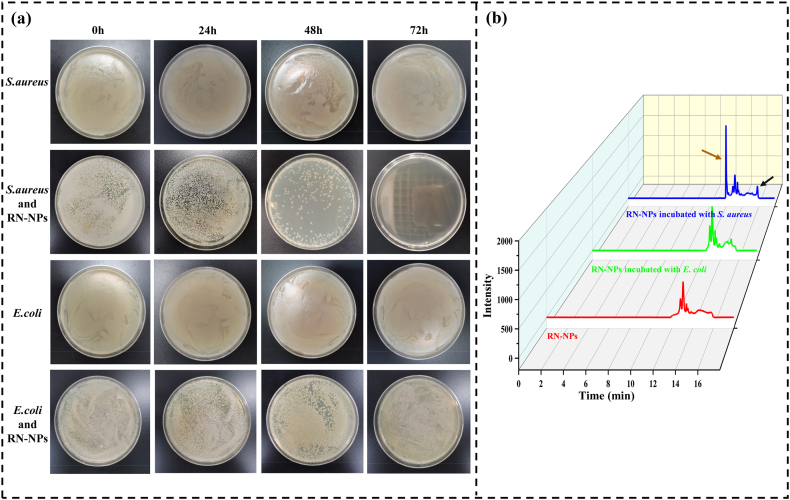
(A) The plate colonies of RN-NPs after incubation with MRSA or *E. coli*; (B) High-performance liquid chromatograms of RN-NPs (red), RN-NPs incubated with *E. coli* (green), and RN-NPs incubated with MRSA (blue), where the brown arrow points to the Res characteristic peak and the black arrow points to the Nisin characteristic peak.

The plate coating method and HPLC analysis method were used to verify the stimulation response mechanism of pore-forming toxin (PFT). In other words, PFT can perforate on the cell membrane, destroy the liposome membrane, and release the RN encapsulated in it. Because *E*. *coli* does not produce PFT, while MRSA can release α-hemolysin (Hla) in the form of soluble monomers. The hydrophobic region of the Hla molecule interacts with phospholipids on the target cell membrane, causing Hla to undergo rapid conformational changes and self-assemble into a multimeric structure. Ultimately, this leads to the formation of transmembrane pores on the cell membrane, resulting in local membrane deformation, perforation, and the release of intracellular contents. According to the study by Markus Schwiering et al., this multimeric structure consists of a triangular arrangement of a cap, rim, and stem, which is crucial for pore formation. Among them, P103C and N105C, located in the triangular region, play an essential role in hemolysis and heptamer formation [45]. Therefore, *E. coli* was selected as the control strain for the experiment. Studies have shown that when the OD 600 nm value of MRSA reaches 0.3, the released α-hemolysin can cause 100 % hemolysis of red blood cells, demonstrating its strong membrane-disrupting ability. Given that α-hemolysin exhibits optimal stability and pore-forming activity at 37 °C and pH 7.4, the α-hemolysin released by MRSA in this experiment should be able to disrupt the liposomal membrane, thereby promoting the leakage of its contents. According to the experimental results, the encapsulation efficiency of Res in RN-NPs is 35.45 %, while that of Nisin is 29.10 %. As shown in Fig. 5a, during the whole experiment, the number of *E. coli* remained stable after incubation with RN-NPs, and there was no significant decrease in the number of *E. coli*, which was consistent with the results of the blank control group. This indicated that the liposomes in the control group were not activated to release Res and Nisin. This is because *E. coli* cannot secrete PFT to form pores on the liposomal membrane, preventing the “intelligent” release of RN from the liposome. In contrast, the number of MRSA in the presence of RN-NPs continuously decreased as the reaction time extended. When MRSA reacted with RN-NPs liposome for 72 h, there was no colony on the plate. This suggests that once α-hemolysin is inserted into the bilayer of the liposome membrane, a pore structure will be formed on the membrane surface, and RN will be released from the liposome and react with MRSA. According to the study by Masashi Watanabe et al., when multilamellar liposomes are exposed to α-hemolysin at concentrations ranging from 1 to 8 μg/mL, liposomes composed of phospholipids and cholesterol bind to α-hemolysin in a dose-dependent manner, leading to significant content release. This suggests that choline-containing phospholipids may be the key components responsible for toxin-membrane interactions that cause membrane damage [20,45,46]. Additionally, cholesterol may further enhance the interaction between α-hemolysin and the liposomal membrane. Since the lipid membrane of RN-NPs is primarily composed of soybean lecithin and cholesterol, with phosphatidylcholine being a major component of soybean lecithin, RN-NPs exhibit a strong binding affinity to α-hemolysin, thereby facilitating effective drug release. The experimental results also accurately prove that a-hemolysin can be combined with RN-NPs to establish an “intelligent” trigger mechanism, to achieve the purpose of α-hemolysin stimulating RN-NPs to respond and release RN.

As mentioned above, α-hemolysin reacts with liposomes to form channels on the liposome membrane, and RN is released from the liposome. Utilizing this feature, the reaction solutions of *E. coli* and MRSA with RN-NPs were collected and analyzed by HPLC. If Res and Nisin were present, the stimulation release mechanism could be proved. As shown in Fig. 5b, there was no chromatographic peak of Res and Nisin in the chromatogram of blank liposomes, which indicated that there was no free Res and Nisin in the suspension. This may be because the HPLC used in this study was not sensitive and the detection limit was high. Similar to this result, Res and Nisin were not detected in the reaction solution of *E. coli* with RN-NPs, as *E. coli* itself does not produce PFT, and therefore cannot trigger the intelligent release of Res and Nisin from the liposome. However, contrary to these results, the presence of Res (brown arrow) and Nisin (black arrow) was detected in the reaction solution of MRSA with RN-NPs, indicating that the a-hemolysin of MRSA can stimulate RN-NPs to release Res and Nisin.

### 3.5 Anti-inflammatory activity *in vitro*

**Fig. 6:**
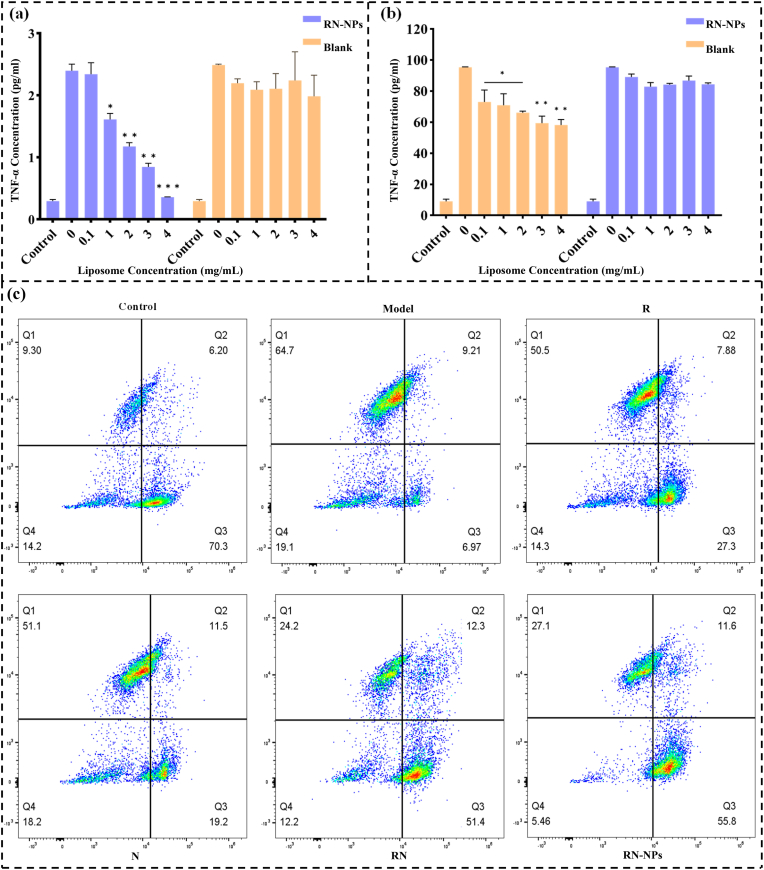
Changes in protein content (A) and TNF-α content (B) after co-incubation of RN-NPs or blank nanoliposomes with LPS-stimulated J774A.1 cells; (C) Flow cytometry assessment of cellular ROS clearance and apoptosis.

In order to further analyze the anti-inflammatory effect of RN-NPs, the anti-inflammatory activity was evaluated *in vitro* using J774A.1 mouse macrophage (Fig. 6). In the model group, LPS stimulation triggered the synthesis and release of a large amount of inflammation-related proteins in J774A.1 cell, which indicated that the modeling method was successful and suitable for subsequent experiments. After co-culturing different concentrations of blank nanoliposomes with LPS-stimulated J774A.1 cells, it was found that the protein content did not decrease significantly, indicating that blank nanoliposomes did not have anti-inflammatory effects (Fig. 6a). In contrast, the protein content produced by J774A.1 cells after RN-NPs treatment decreased significantly with the increase in RN-NPs concentration. When the RN-NPs concentration was increased to 4 mg/mL, the protein content decreased by approximately 82 % compared to the blank nanoliposome group (Fig. 6a). This result demonstrates that RN-NPs exhibit effective anti-inflammatory activity *in vitro*.

TNF-α is one of the main pro-inflammatory factors produced after LPS stimulation. Therefore, to further verify the anti-inflammatory activity of RN-NPs, the expression of TNF-α was analyzed. As shown in Fig. 6b, the TNF-α levels in J774A.1 cells were significantly elevated after LPS stimulation in the model group, which corresponded to the synthesis and release of a large number of inflammation-related proteins in J774A.1 cell induced by LPS stimulation described above. After incubation with blank nanoliposomes and RN-NPs, it was found that the blank liposomes had almost no effect on the change of TNF-α level, further confirming that the liposomes themselves did not possess anti-inflammatory properties. In the RN-NPs group, the TNF-α levels decreased significantly as the concentration of RN-NPs increased, which aligns with previous findings and demonstrates that RN-NPs can effectively block the inflammation amplification mediated by pro-inflammatory cytokines.

LPS induces excessive production of pro-inflammatory mediators and ROS in both immune and non-immune cells [47]. This is characterized by the upregulation of pro-inflammatory cytokines (e.g., TNF-α, IL-6, IL-1β), inflammation-related enzymes (e.g., iNOS, COX-2), ROS generation, inflammasome activation, and the regulation of key signaling pathways such as NF-κB and MAPK [48]. Previous experiments confirmed that LPS stimulation triggers J774A.1 cells to synthesize and release large amounts of inflammation-related proteins and TNF-α. Oxidative stress, characterized by high ROS levels, can cause molecular damage to cellular structures. When ROS production exceeds the cellular antioxidant capacity, it is closely associated with the onset and progression of various diseases [49]. Hydrogen peroxide (H_2_O_2_) induces substantial ROS production in cells, leading to cellular damage and even cell death. To further verify the anti-inflammatory activity of RN-NPs, we analyzed their ROS-scavenging and anti-apoptotic effects. As shown in Fig. 6C, the cell death rate in the model group reached 64.7 %. Compared to the model group, RN-NPs significantly reduced H_2_O_2_-induced apoptosis in J774A.1 cells, exhibiting higher activity than R, N, and blank nanoliposomes. In conclusion, RN-NPs demonstrated strong ROS-scavenging and anti-apoptotic bioactivity *in vitro*, providing valuable data support for subsequent *in vivo* experiments.

In recent years, several studies have confirmed the anti-inflammatory effects of Res. For example, Chen et al. [50]. explored the anti-inflammatory and antiviral mechanisms of Res, and the final results showed that Res could block the secretion of inflammatory factors by regulating the cascade of NF-κB signaling. At the same time, they believed that the NF-κB signaling pathway is the key pathway for Res to exert anti-inflammatory effects. In addition, this conclusion has been confirmed in the study of Mayangsari and Suzuki [51]. Likewise, the anti-inflammatory effect of Nisin has also been confirmed by a large number of studies. Recently, a study investigated the therapeutic mechanism of Nisin Z in treating mastitis and found that Nisin Z could effectively inhibit the activation of the ERK1/2 and p38 mitogen-activated protein kinase (MAPK) signaling pathways, reducing the release of pro-inflammatory cytokines such as TNF-α and IL-1β [52]. Additionally, Han et al. also studied the synergistic antibacterial and anti-inflammatory effects of Nisin combined with lactic acid *in vitro*, and the results showed that the combination of nisin and lactic acid could significantly reduce the mRNA levels of *Helicobacter pylori* bacterial toxins and pro-inflammatory cytokines in human gastric (AGS) cells infected with *H. pylori* [53].

All in all, RN-NPs exhibit significant anti-inflammatory effects *in vitro*, which can effectively inhibit the synthesis of related proteins in J774A.1 cell after LPS stimulation and significantly reduce the secretion of pro-inflammatory factor TNF-α. This finding provides strong experimental support for RN-NPs as a potential anti-inflammatory treatment and lays a foundation for its further application research.

### 3.6 Verification of anti-inflammatory activity in mice

**Fig. 7:**
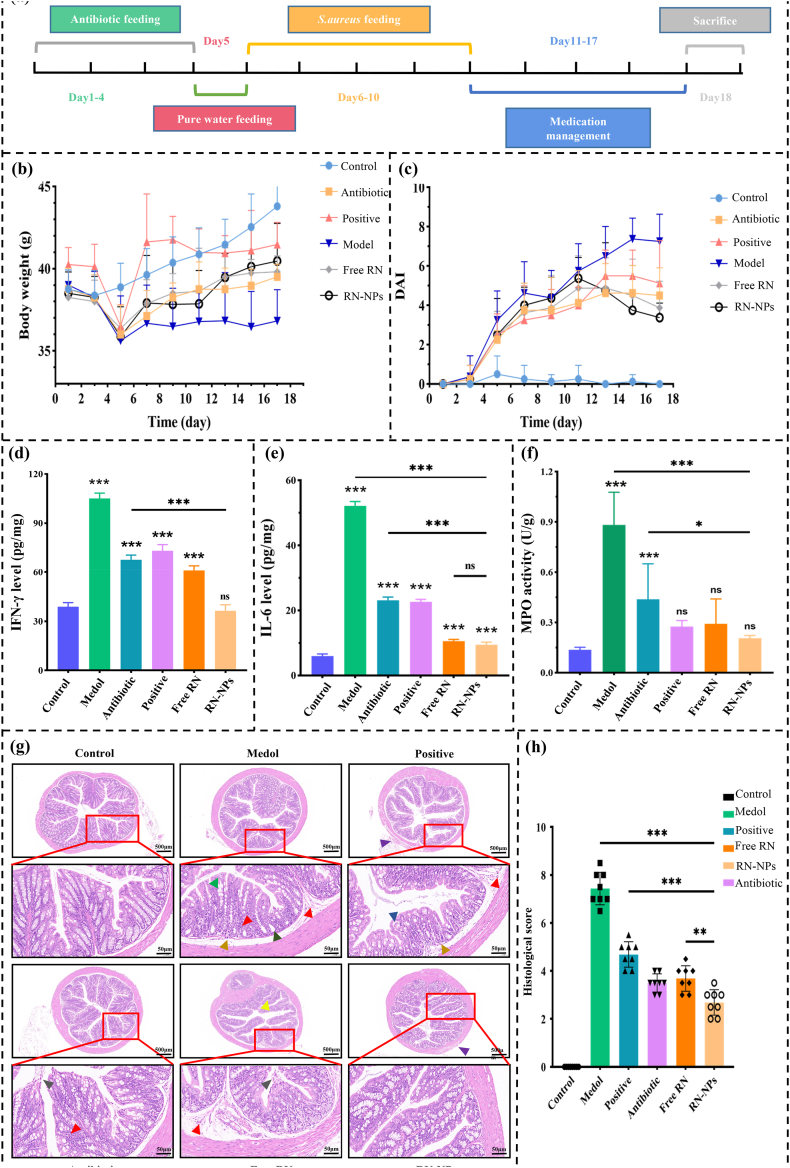
The in vivo efficacy of RN-NPs on MRSA enteritis in mice. (A) Modeling and experimental scheme of RN-NPs in the treatment of MRSA enteritis in mice. After oral administration of antibiotic mixed solution, positive drug oxacillin sodium, free RN or RN-NPs, the body weight (B), disease activity index (DAI) score (C), inflammatory factors INF-γ (D) and IL-6 (E) and MPO activity in colon tissue were measured. (F) To evaluate the therapeutic effect of RN-NPs on enteritis (n = 8). Hematoxylin and eosin staining (H&E) sections (G) and histological scores (H) of colon tissues of mice in the blank group, model group, positive control group, antibiotic group, free RN group, and RN-NPs group. Green arrow: Occasional edema; Brown arrow: Intestinal gland dilation; Orange arrow: Granulocytes; Red arrow: Lymphocyte infiltration; Dark gray arrows: Mucosal epithelial cell necrosis or shedding; Purple arrows: Muscle cell arrangement.

The efficacy and potential of RN-NPs in the targeted treatment of MRSA enteritis in mice were investigated by establishing an antibiotic-induced MRSA enteritis model. The modeling and experimental design for RN-NPs treatment of MRSA enteritis in mice are shown in Fig. 7a. Changes in body weight in mice serve as key biological indicators that indirectly reflect disease progression and treatment efficacy (Fig. 7b). In this experiment, the body weight of healthy mice in the blank group gradually increased throughout the experiment, while all mice fed with the antibiotic mixture showed a decrease in body weight. After the first day of inoculation with MRSA, the body weight of the mice in each experimental group increased, which may be because the number of MRSA colonization was not enough to cause a significant disease response. After drug treatment, the body weight of mice in all experimental groups showed a recovery trend. Among them, the mice in the positive drug group (oxacillin sodium) demonstrated a relatively slower recovery of body weight. Although their weight increased after stopping antibiotic feeding and MRSA inoculation, the increase was slow, indicating that the positive drug had a delayed effect and only general efficacy in treating MRSA enteritis. In contrast, the body weight of mice treated with free RN or RN-NPs recovered rapidly, especially in the RN-NPs group, which proved that RN-NPs could play a role faster and had the best recovery effect.

The disease activity index (DAI) of mice was evaluated by comprehensive analysis of body weight changes, fecal properties, and fecal occult blood indicators. As shown in Fig. 7c, the DAI score of the enteritis model group maintained an upward trend, indicating that the severity of enteritis was increasing. With the prolongation of administration time, the DAI scores of mice in other groups decreased. It is worth mentioning that although positive drugs have shown some efficacy in the treatment, there is a delay in their effect, which is consistent with our previous research conclusions. The DAI scores of the free RN group and the RN-NPs group began to decrease significantly from the second day of administration, especially in the RN-NPs group. This suggests that RN-NPs have a stronger therapeutic effect compared to free RN and the positive drug group. In summary, compared to the positive drug treatment group and the free RN group, RN-NPs not only facilitated faster weight recovery in mice but also significantly reduced DAI scores, demonstrating its potent anti-inflammatory effects and better clinical application potential.

Interferon-gamma (IFN-γ) and interleukin-6 (IL-6) are important cytokines that play crucial roles in the immune system. Among them, IFN-γ, secreted by T cells and natural killer cells, activates macrophages and regulates immune responses [54]. When inflammation occurs, the level of IFN-γ will increase, enhancing the ability of antigen presentation and killing pathogens. IL-6, a pro-inflammatory cytokine, promotes the differentiation of B cells and T cells and plays a critical role in chronic inflammation, with its levels typically correlating directly with the severity of inflammation [55]. As shown in Fig. 7d and e, the levels of IFN-γ and IL-6 in the antibiotic-treated mice were higher than in normal mice but significantly lower than in the model group. This phenomenon may be due to MRSA colonization, which exacerbated the severity of inflammation in the mice. After drug treatment, the levels of IFN-γ and IL-6 in mice decreased. Specifically, the inhibitory effects of free RN and RN-NPs at a concentration of 4 mg/mL were significantly better than those of the positive control group. Compared with the free RN group, RN-NPs treatment could significantly reduce the expression levels of inflammatory cytokines IFN-γ and IL-6. This further proves the advantages of RN-NPs in anti-inflammation.

Myeloperoxidase (MPO), an enzyme that plays a role in host defense, is a marker of neutrophil infiltration [56]. It can be seen from Fig. 7f that the MPO activity of healthy mice in the blank group was within the normal range, but the significant increase of MPO activity in the model group indicated the production of inflammation. Compared with the model group, the positive control group, free RN group, and RN-NPs group significantly inhibited the increase of MPO activity. At the end of treatment, the MPO content in the RN-NPs group decreased by about 52 % and 28 %, respectively, compared with the positive control group and the free RN group. It can be seen that RN-NPs showed significant anti-inflammatory effects in reducing the levels of inflammatory cytokines IFN-γ and IL-6 and inhibiting MPO activity, which further proved its potential as an anti-inflammatory therapeutic agent.

H&E staining was used to observe the effect of RN-NPs on the colonic tissue morphology of mice, and a histological scoring of disease severity was performed on the colon tissue (Fig. 7g and h) [57]. In the normal group, healthy mice had abundant intestinal folds, a mucosal epithelium with tightly arranged intestinal glands in the lamina propria, numerous goblet cells, loose connective tissue in the submucosa, and clearly defined muscular layers. In the model group, occasional edema was observed in the intestinal mucosal epithelial cells (green arrow), slight dilation of the intestinal glands (brown arrow), and significant infiltration of granulocytes and lymphocytes in the lamina propria (orange arrow and red arrow). Similarly, in the antibiotic group, necrosis and shedding of mucosal epithelial cells (dark gray arrow) and slight infiltration of granulocytes in the lamina propria (red arrow) were observed. However, the intestinal inflammation of mice was alleviated after drug treatment. Among them, compared with the model group, the symptoms of intestinal inflammation in the positive control group were slightly alleviated, but a small amount of lymphocyte and granulocyte infiltration (red arrow and orange arrow) were still visible. The mucosal epithelium was separated from the lamina propria (blue arrows), and a small range of muscle cells were loosely arranged in the muscle layer (purple arrow). Notably, in the free RN group, small amounts of necrotic mucosal epithelial cells were found to be necrotic and exfoliated in the intestinal lumen, along with focal aggregation (dark gray arrow) and infiltration of lymphocytes (red arrow), indicating the limited therapeutic effect of free RN on intestinal inflammation. In the RN-NPs group, there were abundant intestinal folds, increased crypt depth, and reduced distance between the crypt base and muscularis mucosa, with numerous goblet cells and a large number of intestinal glands in the lamina propria, and increased intracellular mucus. Although localized loosening of muscle cells in the muscularis (purple arrow) was still observed, there was no significant histological damage. Furthermore, the histological score of the RN-NPs group decreased most significantly, demonstrating that RN-NPs had a remarkable therapeutic effect on MRSA enteritis.

### 3.7 Biocompatibility evaluation of RN-NPs

**Fig. 8:**
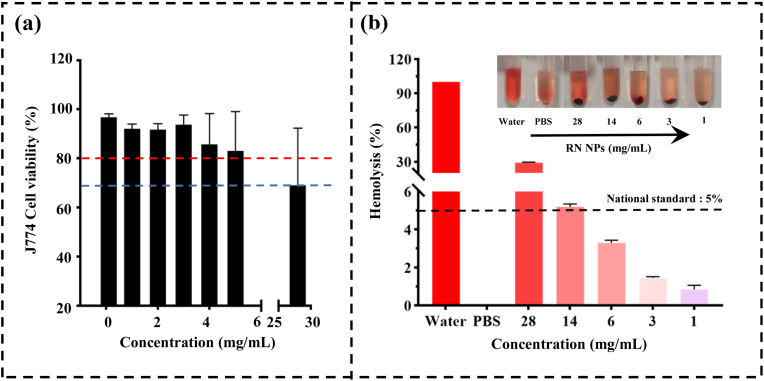
(A) The cell survival rate of RN-NPs after incubation with J774A.1 mouse macrophage; (B) Hemolytic experiment of RN-NPs and mouse blood.

To confirm the biosafety of RN-NPs, an MTT assay was conducted to evaluate the cytotoxicity of RN-NPs on J774A.1 mouse macrophage (Fig. 8a). Across all tested concentration ranges, the cell survival rate after RN-NPs treatment was above 70 %. According to ISO 10993–5:2009 (Biological Evaluation of Medical Devices—Part 5: Tests for *In vitro* Cytotoxicity), which suggests a cytotoxicity threshold of 70 % [58], this indicates that RN-NPs exhibit minimal cytotoxic effects on J774A.1 cells. This may be related to the inherent properties of Res and Nisin. Res, as a natural dietary polyphenol, provides significant health benefits to the gut microbiota. These beneficial effects are mainly related to their physiological activities in the gastrointestinal tract (GI). After ingestion, Res is rapidly metabolized by host and gut microbial enzymes, producing glucuronidated and sulfated metabolites. In particular, resveratrol-3-O-sulfate has outstanding regulatory functions on the growth of gut microbiota and intestinal barrier function [59]. Nisin is degraded by digestive enzymes in the body without producing toxic substances, and it does not significantly negatively impact the gut microbiota. Moreover, Nisin has been approved as a food additive in many countries due to its safety and potent antibacterial activity. It can be seen that RN-NPs demonstrate strong biosafety.

Oral medications generally enter the bloodstream through the capillaries in the small intestine. Therefore, the biocompatibility of RN-NPs was further evaluated by hemolytic experiments. As shown in Fig. 8b, water causes hemolysis with a hemolysis rate as high as 100 %, while PBS does not induce hemolysis. This has also been confirmed in Huang et al.’s research. They evaluated the biocompatibility of nanoparticles (CABBR-NPs) formed by the self-assembly of berberine and cinnamic acid, and the results showed that the hemolysis rates of water and PBS were 100 % and 0 %, respectively. Additionally, CABBR-NPs did not cause significant hemolysis in rat red blood cells [22]. We observed in Fig. 8b that when the concentration of RN-NPs was in the range of 0–6 mg/mL, the hemolysis rate caused by RN-NPs was lower than the internationally recognized standard of 5 %. This indicates that the blood of mice treated with RN-NPs has no obvious hemolysis and has good biocompatibility. Previous studies have also supported this conclusion. Comparing the hemolysis of free Res with that of Res embedded in liposomes, it was found that free Res could cause hemolysis of red blood cells, while no obvious hemolysis was observed after Res embedded in liposomes was incubated with human red blood cells for 6 h [60]. Thus, RN-NPs exhibit excellent biocompatibility and hold great potential for subsequent clinical applications.

### 3.8 Safety evaluation of animal experiments

**Fig. 9:**
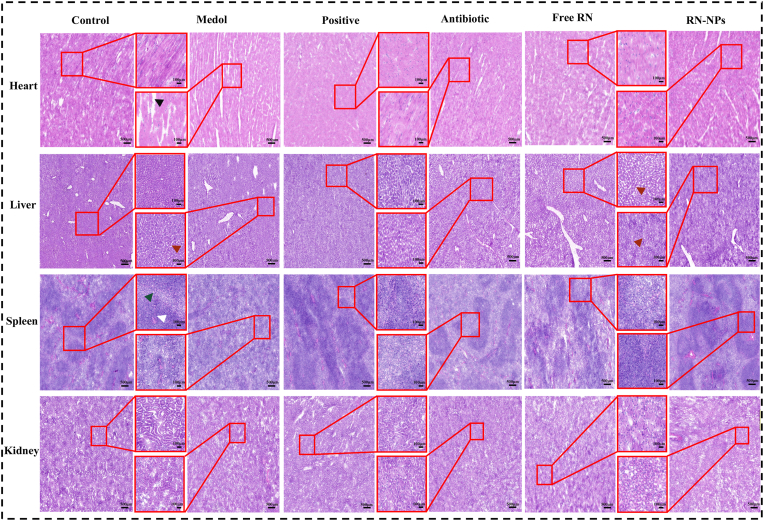
In vivo safety evaluation of RN-NPs in MRSA enteritis test. H&E histological sections of the heart, liver, spleen, and kidney of mice in the control group, model group, positive control group, antibiotic group, free RN group, and RN-NPs group ( × 2.5 and × 12).

Given the good biocompatibility and significant anti-inflammatory effects of RN-NPs, further evaluations were conducted to explore whether RN-NPs treatment would affect the major organs of mice (Fig. 9). In this study, analysis of heart tissue sections revealed that in the control group (healthy mice), the myocardial fibers were evenly stained, cell boundaries were distinct, fibers were uniformly arranged, the striations of myocardial cells were clear, and no abnormalities were observed in the stroma. However, the myocardial cells in the model group showed more obvious pathological features, with black arrows indicating cell swelling, increased intercellular spaces, and loosened cytoplasm with lighter staining. In contrast, this phenomenon was alleviated after drug treatment. In particular, the therapeutic effect of RN-NPs is the most prominent. It is evident that the myocardial cells were placed more neatly, and the myocardial tissue's overall compactness was recovered. At the same time, through the observation of liver tissue, it was found that compared with the normal liver of the blank group (healthy mice), the gap between the hepatocytes in the model group became larger (red-brown arrow), and the liver appeared edema. However, the recovery of liver sinusoids was not significant after treatment with the positive drug or free RN. In the RN-NPs treatment group, it was found that the hepatocyte gap was significantly improved and the edema phenomenon was also alleviated. In the histological analysis of the spleen, the spleen of healthy mice showed clear boundaries between the white pulp and red pulp (indicated by white and dark green arrow), distinct marginal zones, and intact lymphoid follicles. In the model group, the white pulp structure of the spleen tissue was damaged and the marginal area became blurred. After drug treatment, the structural damage of the spleen was improved, especially in the RN-NPs treatment group. In the observation of kidney tissue, no obvious damage or lesions were observed in any group, indicating that the kidney was not significantly affected under the experimental conditions. In summary, RN-NPs not only showed no adverse effects on other organs in the treatment of enteritis in mice but also had significant effects in protecting the myocardium, liver, and spleen.

## 4 Conclusions

In conclusion, this study developed a novel and safe passive-targeted drug delivery system (RN-NPs) for the treatment of MRSA enteritis. RN-NPs effectively protected Res and Nisin from the degradation of gastric acid and protease, avoiding the premature release of drugs in the digestive tract. At the same time, the unique phospholipid bilayer structure of RN-NPs could specifically react with the pore-forming toxin released by MRSA to form a pore structure, so that the drug could achieve targeted release in the intestinal inflammatory site. As a result, Res and Nisin are used more effectively, and the treatment's safety and effectiveness are greatly increased while the negative effects of non-targeted medicines are decreased. All things considered, RN-NPs exhibit considerable promise as an oral targeted drug delivery technology, offering a workable, safe, and efficient treatment for MRSA enteritis. Various researchers in the domains of microbiology, chemistry, biomaterials science, and nanomedicine may be interested in this work.

## CRediT authorship contribution statement

**Qingli Yang:** Writing – original draft, Conceptualization. **Jindi Wang:** Writing – original draft, Conceptualization. **Pengdong Sun:** Resources, Methodology. **Fangyuan Zhao:** Software, Investigation. **Jian Ju:** Writing – review & editing, Conceptualization.

## Declaration of competing interest

The authors declare that they have no known competing financial interests or personal relationships that could have appeared to influence the work reported in this paper.

## Data Availability

Data will be made available on request.

## References

[1] Yang P., Huo Y., Yang Q., Zhao F., Li C., Ju J. (2025). Synergistic anti-biofilm strategy based on essential oils and its application in the food industry. World J. Microbiol. Biotechnol..

[2] Wong Y.Y., Chow Y.L. (2024). Exploring the potential of spice‐derived phytochemicals as alternative antimicrobial agents. eFood.

[3] Larcombe S., Jiang J.-H., Hutton M.L., Abud H.E., Peleg A.Y., Lyras D.J.J.o.M.M. (2020). A mouse model of Staphylococcus aureus small intestinal infection. J. Med. Microbiol..

[4] Cabezas‐Mera F., Cedeño‐Pinargote A.C., Tejera E., Álvarez‐Suarez J.M., Machado A. (2024). Antimicrobial activity of stingless bee honey (Tribe: Meliponini) on clinical and foodborne pathogens: a systematic review and meta‐analysis. Food Frontiers.

[5] Wang Y., Yang Q., Zhao F., Li M., Ju J. (2024). Synergistic antifungal mechanism of eugenol and citral against Aspergillus niger: molecular Level. Ind. Crop. Prod..

[6] Zeng L., Fan A., Yang G., Nong Y., Lu Y., Yang R. (2024). Nisin and ε-polylysine combined treatment enhances quality of fresh-cut jackfruit at refrigerated storage. Front. Nutr..

[7] Gough R., O'Connor P.M., Rea M.C., Gómez-Sala B., Miao S., Hill C., Brodkorb A.J.L. (2017). Simulated gastrointestinal digestion of nisin and interaction between nisin and bile. Lebensm. Wiss. Technol..

[8] Xie Z., Chen X.J.F.R.I. (2023). Healthy benefits and edible delivery systems of resveratrol: a review. Food Rev. Int..

[9] Dikmetas D.N., Yenipazar H., Karaca A.C. (2024). Recent advances in encapsulation of resveratrol for enhanced delivery. Food Chem..

[10] Todan L., Voicescu M., Culita D.C., Pandele-Cuşu J., Albu C., Kuncser A.C.J.C.P. (2021). Ecological formulation for improving resveratrol stability and release in aqueous environment. Chem. Pap..

[11] Gao Q., Feng Z., Wang Z., Zhao F., Ju J. (2025). Carvacrol induces apoptosis in *Aspergillus niger* through ROS burst. World J. Microbiol. Biotechnol..

[12] Feng Z., Zhang Q., Wang Y., Yang Q.L., Zhao F., Ju J. (2025). Anti-*Aspergillus niger* mechanism of small molecular combinations of essential oils and their application in extending the shelf-life of bread. Food Biosci..

[13] Zhang Q., Huo Y., Yang Q., Zhao F., Li M., Ju J. (2025). Migration of chemical substances from packaging materials to food. Food Chem..

[14] Ni W., Wei F., Sun C., Yao J., Zhang X., Zhang G. (2024). Inhibitory effect of Jingfang mixture on Staphylococcus aureus α-hemolysin. Microb. Pathog..

[15] Huang R., Yao A., Yan Y., Wang J., Li Q., Li K., Wu J. (2024). Development and characterization of multifunctional fish gelatin composite films reinforced with ε‐polylysine and zinc oxide nanoparticles. efood.

[16] Huang X., Liu T., Zhou C., Huang Y., Liu X., Yuan H.J.A. (2021). Antifungal activity of essential oils from three Artemisia species against Colletotrichum gloeosporioides of mango. Antibiotics.

[17] Liu W., Hou Y., Jin Y., Wang Y., Xu X., Han J.J.T.i.F.S. (2020). Technology. Research progress on liposomes: application in food, digestion behavior and absorption mechanism. Trends Food Sci. Technol..

[18] Liu W., Liu J., Salt L.J., Ridout M.J., Han J., Wilde P.J. (2019). Structural stability of liposome-stabilized oil-in-water pickering emulsions and their fate during *in vitro* digestion. Food Funct..

[19] Hu Q., Zhou F., Ly N.K., Ordyna J., Peterson T., Fan Z., Wang S.J. A.n. (2023). Development of multifunctional nanoencapsulated trans-resveratrol/chitosan nutraceutical edible coating for strawberry preservation. ACS Nano.

[20] Tang C., Li Q., Lin T. (2021). Lycopene attenuates Staphylococcus aureus-induced inflammation via inhibiting α-hemolysin expression. Microb. Infect..

[21] Shrestha N., Xu Y., Prévost J.R., McCartney F., Brayden D., Frédérick R., Beloqui A., Préat V.J. A.b. (2022). Impact of PEGylation on an antibody-loaded nanoparticle-based drug delivery system for the treatment of inflammatory bowel disease. Acta Biomater..

[22] Huang X., Wang P., Li T., Tian X., Guo W., Xu B., Huang G., Cai D., Zhou F., Zhang H. (2019). Self-assemblies based on traditional medicine berberine and cinnamic acid for adhesion-induced inhibition multidrug-resistant Staphylococcus aureus. ACS Appl. Mater. Interfaces.

[23] Hu R., Yang T., Ai Q., Shi Y., Ji Y., Sun Q., Wang Z. (2024). Autoinducer-2 promotes the colonization of Lactobacillus rhamnosus GG to improve the intestinal barrier function in a neonatal mouse model of antibiotic-induced intestinal dysbiosis. J. Transl. Med..

[24] Lin W., Chen H., Chen X., Guo C. (2024). The roles of neutrophil-derived myeloperoxidase (MPO) in diseases: the new progress. Antioxidants.

[25] Ye N., Zhao P., Ayue S., Qi S., Ye Y., He H., Dai L., Luo R., Chang D., Gao F. (2023). Folic acid-modified lactoferrin nanoparticles coated with a laminarin layer loaded curcumin with dual-targeting for ulcerative colitis treatmen. Int. J. Biol. Macromol..

[26] Zhao S., Zhang J., Qiu M., Hou Y., Li X., Zhong G., Gou K., Li J., Zhang C., Qu Y. (2024). Mucoadhesive and thermosensitive Bletilla striata polysaccharide/chitosan hydrogel loaded nanoparticles for rectal drug delivery in ulcerative colitis. Int. J. Biol. Macromol..

[27] Malaguarnera L. (2019). Influence of resveratrol on the immune response. Nutrients.

[28] Varoni E.M., Lo Faro A.F., Sharifi-Rad J., Iriti M. (2016). Anticancer molecular mechanisms of resveratrol. Front. Nutr..

[29] Meng T., Xiao D., Muhammed A., Deng J., Chen L., He J. (2021). Anti-inflammatory action and mechanisms of resveratrol. Molecules.

[30] Haider T., Pandey V., Behera C., Kumar P., Gupta P.N., Soni V. (2022). Nisin and nisin-loaded nanoparticles: a cytotoxicity investigation. Drug Dev. Ind. Pharm..

[31] Liu L., Ingmer H., Vestergaard M. (2021). Genome-wide identification of resveratrol intrinsic resistance determinants in Staphylococcus aureus. Antibiotics.

[32] Elsherif W.M., Hassanien A.A., Zayed G.M., Kamal S.M. (2024). Natural approach of using nisin and its nanoform as food bio-preservatives against methicillin resistant Staphylococcus aureus and *E. coli* O157: H7 in yoghurt. BMC Vet. Res..

[33] Prevete G., Simonis B., Mazzonna M., Mariani F., Donati E., Sennato S., Ceccacci F., Bombelli C. (2023). Resveratrol and resveratrol-loaded galactosylated liposomes: anti-adherence and cell wall damage effects on staphylococcus aureus and MRSA. Biomolecules.

[34] Liu L., Ingmer H., Vestergaard M. (2021). Genome-wide identification of resveratrol intrinsic resistance determinants in Staphylococcus aureus. Antibiotics.

[35] Li Y., Sun K., Chen S., Zhao J., Lei Y., Geng L. (2023). Nano-resveratrol liposome: physicochemical stability, in vitro release, and cytotoxicity. Appl. Biochem. Biotechnol..

[36] Wu C., Zhi Z., Duan M., Sun J., Jiang H., Pang J. (2023). Insights into the formation of carboxymethyl chitosan-nisin nanogels for sustainable antibacterial activity. Food Chem..

[37] Gulzar S., Tagrida M., Prodpran T., Benjakul S. (2022). Antimicrobial film based on polylactic acid coated with gelatin/chitosan nanofibers containing nisin extends the shelf life of Asian seabass slices. Food Packag. Shelf Life.

[38] Anderson M., Omri A. (2004). The effect of different lipid components on the in vitro stability and release kinetics of liposome formulations. Drug Deliv..

[39] Santhosh P.B., Velikonja A., Perutkova Š., Gongadze E., Kulkarni M., Genova J., Ulrih N.P. (2014). Influence of nanoparticle–membrane electrostatic interactions on membrane fluidity and bending elasticity. Chem. Phys. Lipids.

[40] Li X., Wang X., Zhang H., Gong L., Meng X., Liu B. (2023). OSA-starch stabilized EPA nanoliposomes: preparation, characterization, stability and digestion in vitro and *in vivo*. Food Chem..

[41] Luo Y., Wang F., Yuan X., Wang K., Sun Q., Wang H., Pu C., Tang W. (2022). Walnut peptide loaded proliposomes with hydroxyapatite as a carrier: fabrication, environmental stability, and in vitro digestion attribute. Food Rev. Int..

[42] Mei L., Liao K., Chen H., Zhang Y., Zhang Z., Li Q., Li M. (2024). Application of nanomaterials and related drug delivery systems in autophagy. Molecules.

[43] Li Z., Zhu Y., Zeng H., Wang C., Xu C., Wang Q., Wang H., Li S., Chen J., Xiao C. (2023). Mechano-boosting nanomedicine antitumour efficacy by blocking the reticuloendothelial system with stiff nanogels. Nat. Commun..

[44] Wang K., Chen D., Zhang C., Lu L., Shang F., Li Y. (2024). Polyethylene glycol-modified cationic liposome as a promising Nano spray for Acute Pneumonia treatment. Polymers.

[45] Du Y., Liu L., Zhang C., Zhang Y. (2018). Two residues in Staphylococcus aureus α-hemolysin related to hemolysis and self-assembly. Infecti. drug resist.

[46] Wolfmeier H., Mansour S.C., Liu L.T., Pletzer D., Draeger A., Babiychuk E.B., Hancock R.E. (2018). Liposomal therapy attenuates dermonecrosis induced by community-associated methicillin-resistant Staphylococcus aureus by targeting α-type phenol-soluble modulins and α-hemolysin. EBioMedicine.

[47] Li X., Zhang X., Kang Y., Cai M., Yan J., Zang C., Qi Y. (2024). Scutellarein suppresses the production of ROS and inflammatory mediators of LPS-activated bronchial epithelial cells and attenuates acute lung injury in mice. Antioxidants.

[48] Camba-Gómez M., Gualillo O., Conde-Aranda J. (2021). New perspectives in the study of intestinal inflammation: focus on the resolution of inflammation. Int. J. Mol. Sci..

[49] Sousa N.A., Oliveira G.A., de Oliveira A.P., Lopes A.L.F., Iles B., Nogueira K.M., Medeiros J.V.R. (2020). Novel ocellatin peptides mitigate LPS-induced ROS formation and NF-kB activation in microglia and hippocampal neurons. Sci. Rep..

[50] Chen X., Song X., Zhao X., Zhang Y., Wang Y., Jia R., Zou Y., Li L., Yin Z. (2022). Insights into the anti‐inflammatory and antiviral mechanisms of resveratrol. Mediat. Inflamm..

[51] Mayangsari Y., Suzuki T. (2018). Resveratrol ameliorates intestinal barrier defects and inflammation in colitic mice and intestinal cells. J. Agric. Food Chem..

[52] Huang F., Teng K., Liu Y., Wang T., Xia T., Yun F., Zhong J. (2022). Nisin Z attenuates lipopolysaccharide-induced mastitis by inhibiting the ERK1/2 and p38 mitogen-activated protein kinase signaling pathways. J. Dairy Sci..

[53] Han S.G., Kwon H.C., Hong S.J., Han S.G. (2023). In vitro synergistic antibacterial and anti-inflammatory effects of nisin and lactic acid in yogurt against Helicobacter pylori and human gastric cells. Food Sci. Anim. Resour..

[54] Liu W., Zhang S., Wang J. (2022). IFN-γ, should not be ignored in SLE. Front. Immunol..

[55] Rose‐John S. (2022). Local and systemic effects of interleukin‐6 (IL‐6) in inflammation and cancer. FEBS Lett..

[56] Zheng R., Moynahan K., Georgomanolis T., Pavlenko E., Geissen S., Mizi A., Grimm S., Nemade H., Rehimi R., Bastigkeit J.J.I. (2024). Remodeling of the endothelial cell transcriptional program via paracrine and DNA-binding activities of MPO. iScience.

[57] Heavey M.K., Hazelton A., Wang Y., Garner M., Anselmo A.C., Arthur J.C., Nguyen J.J.N.C. (2024). Targeted delivery of the probiotic Saccharomyces boulardii to the extracellular matrix enhances gut residence time and recovery in murine colitis. Nat. Commun..

[58] Rekha S., Anila E. (2019). In vitro cytotoxicity studies of surface modified CaS nanoparticles on L929 cell lines using MTT assay. Mater. Lett..

[59] Zhang B., Xu Y., Lv H., Pang W., Wang J., Ma H., Wang S. (2021). Intestinal pharmacokinetics of resveratrol and regulatory effects of resveratrol metabolites on gut barrier and gut microbiota. Food Chem..

[60] Caddeo C., Pucci L., Gabriele M., Carbone C., Fernàndez-Busquets X., Valenti D., Pons R., Vassallo A., Fadda A.M., Manconi M. (2018). Stability, biocompatibility and antioxidant activity of PEG-modified liposomes containing resveratrol. Int. J. Pharm..

